# Inhibition of protein kinase D disrupts spindle formation and actin assembly during porcine oocyte maturation

**DOI:** 10.18632/aging.101667

**Published:** 2018-12-14

**Authors:** Yu Zhang, Hong-Hui Wang, Xiang Wan, Yao Xu, Meng-Hao Pan, Shao-Chen Sun

**Affiliations:** 1 College of Animal Science and Technology, Nanjing Agricultural University, Nanjing 210095, China

**Keywords:** oocyte, meiosis, spindle, actin, PKD

## Abstract

Protein kinase D (PKD) subfamily which includes PKD1, PKD2 and PKD3 is a novel family of serine/threonine kinases. PKD has been widely implicated in the regulation of multiple physiological effects including immune responses, apoptosis and cell proliferation. However, the roles of PKD in oocytes have not been fully clarified. In this study we investigated the regulatory functions of PKD during porcine oocyte maturation. Our results indicated that PKD expressed in porcine oocytes and the inhibition of PKD family activity led to the failure of meiosis resumption and the first polar body extrusion. Further analysis indicated that the spindle assembly and chromosome alignment were disrupted after PKD family inhibition, and this might be through its regulatory role on MAPK phosphorylation. We also found that PKD phosphorylated cofilin for actin assembly, which further affected cortical actin distribution, indicating the roles of PKD family on cytoskeleton. In addition, a decreased expression of PKD in postovulatory aging porcine oocytes was observed, which might connect PKD with cytoskeleton defects in aged oocytes. Taken together, these results suggest that PKD possesses important functions in porcine oocyte maturation by regulating spindle organization and actin assembly.

## Introduction

Fully grown mammalian oocytes are arrested at the diplotene stage of the first meiotic prophase within ovarian follicles, which is also called germinal vesicle (GV) stage. In order to produce fertilizable female gametes, these oocytes must undergo well-regulated meiotic maturation, including meiosis resumption, proper apparatus assembly and arrangement, as well as first polar body (PBI) extrusion. A combination of cytoskeleton, including microtubules and actin filaments is pivotal for the success meiosis of oocytes. Microtubules form a specialized bipolar-shaped spindle at the center of oocyte at the metaphase I (MI) stage, and the accurate establishment of the meiotic spindle drives the congression of chromosomes at the equatorial plate [1]. Then the inter-chromosomal microtubules form the central spindle to lead chromosomes segregation during anaphase I (AI) in oocytes [2]. Actin filaments accumulated both at the cytoplasm and cortex of oocytes, and are directly involved in pushing meiotic spindle to the cortex during meiosis I, which also maintain asymmetric spindle positioning at metaphase II (MII) [3,4]. At the telophase I (TI) stage, the special actin-based cortical bulges and the meiotic spindle midzone induces the formation of cytokinetic furrows for pinching off a polar body [5–7].

Protein kinase D (PKD) family, which includes three isoforms PKD1, PKD2, and PKD3 [8–10], is a novel family of serine/threonine kinases [9,11]. PKD is the homology with conserved domains of the protein kinase C (PKC) subfamily members. In most cellular systems, PKD family members are activated through PKC-dependent pathway by phosphorylation approach. The PKC-PKD signaling cascade has been increasingly implicated in the regulation of multiple important biological responses including protein transport, cell proliferation and survival [12,13]. PKD has emerged as key regulators of protein transport from the Golgi to the plasma membrane [14,15]. PKD also modulates apoptotic responses in different models [16,17]. Moreover, PKD is activated by oxidative stress and triggers the activation of NF-B signaling, leading to the induction of SOD2 for increased cellular survival in response to oxidative stress [18,19]. Recently, the PKD family is reported to be an important regulator of F-actin remodeling for cell motility [20,21]. Despite of these important discoveries, the functions of PKD in mammalian oocyte meiosis has not been investigated.

In current study, we inhibited PKD family activity to explore the roles of PKD in porcine oocytes and we showed that PKD served as a key regulator for proper spindle organization and actin assembly for polar body extrusion of porcine oocytes. We also provided a potential link for PKD and cytoskeleton defects in aged oocytes.

## RESULTS

### Inhibition of PKD family results in the failure of oocyte maturation

**Figure 1:**
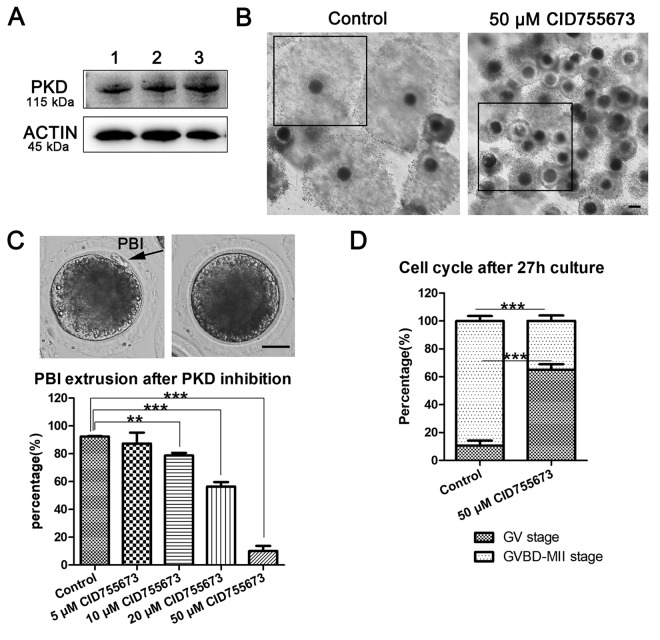
**Effects of PKD inhibition on porcine oocyte maturation.** (**A**) The expression of PKD in porcine oocytes. 1, 2, 3 indicates the samples of three replicates. Rabbit monoclonal anti-PKD antibody was adopted. (**B**) Representative images of cumulus expansion in the control and CID755673-treated groups. Black Crop indicated the status of cumulus expansion in porcine oocytes. Bar = 100 μm (**C**) Rate of oocytes which extruded the PBI in the control and CID755673 treatment groups, respectively. The rate of PBI extrusion was significantly decreased in 10 μM, 20 μM and 50 μM CID755673-exposed groups. Bar = 30 μm. (**D**) The proportions of cell cycle distribution were recorded in the control and CID755673 treatment groups. Data were expressed as mean percentage ± s.d. from at least three independent experiments. **, significant difference (*P* < 0.01), ***, significant difference (*P* < 0.001).

In this study, we first examined the existence of PKD and our results showed that PKD protein expressed in porcine oocytes (Figure 1A). And then we assessed COCs viability and oocytes maturation after PKD inhibition using the PKD specific inhibitor CID755673. After culturing for 44 h following different concentrations of 5 μM, 10 μM, 20 μM and 50 μM CID755673 treatment, we found that the expansion of the peripheral layers cumulus was seriously affected. As shown in Figure 1B, most of the cumulus cells was fully expanded in the control group, whereas partially expanded or not expanded cumulus were observed in 50 μM CID755673 treatment groups. Moreover, CID755673 treatment also disturbed PBI extrusion of porcine oocytes compared with the control groups. The percentage of PBI also confirmed this: as shown in Figure 1C, compared with the control group (92.41 ± 0.77%, n = 276), the PBI extrusion rates were markedly declined after CID755673 treatment in a dose-dependent manner. And the proportion of PBI was 87.37 ± 15.74% (n = 138, *P* > 0.05), 78.77 ± 3.68% (n = 245, *P* < 0.01), 56.42 ± 6.53% (n = 241, *P* < 0.001) and 10.11 ± 7.19% (n = 228, *P* < 0.001) in 5 μM, 10 μM, 20 μM and 50 μM CID755673 groups respectively. The failure of polar body extrusion led us to examine meiosis progression after PKD inhibition. After 27h culture, only 10.66 ± 7.18% (n = 179) of oocytes were arrested at GV stage, while most of 50 μM CID755673-treated oocytes were arrested at GV stage (65.09 ± 7.90%, n = 166, *P* < 0.001) (Figure 1D). These findings imply that PKD inhibition may cause the block of meiosis resumption and the failure of polar body extrusion during porcine oocyte maturation. To analyze the roles of PKD family on oocyte meiosis, a concentration of 20 μM was applied for subsequent studies.

### Inhibition of PKD family affects p-MAPK-mediated spindle organization in porcine oocytes

**Figure 2:**
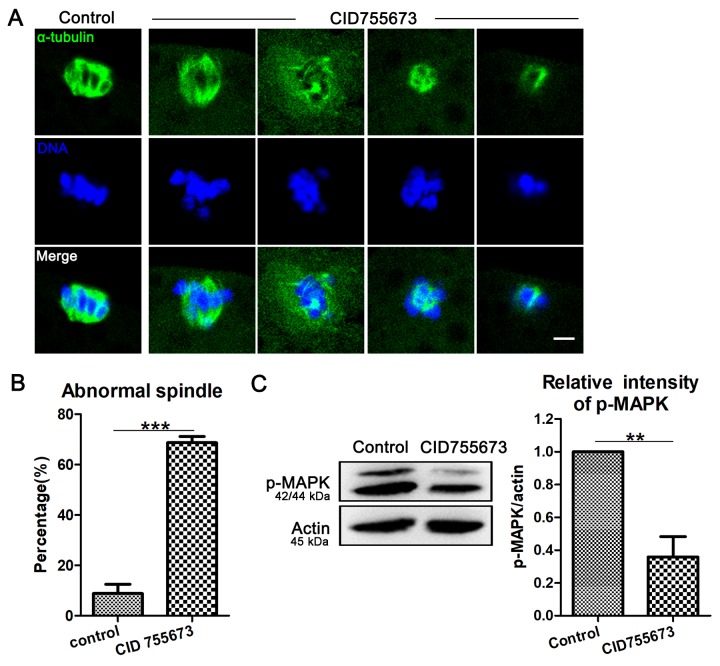
**Effects of PKD inhibition on meiotic spindle configurations and chromosome alignment.** (**A**) Representative images of spindle (green) morphologies and chromosome (blue) alignment in the oocytes from control and CID755673 treatment groups. It showed a typical barrel-shaped spindles and well aligned chromosomes in control oocytes; while, unfocused or tripolar poles spindles with agglutinative/scattered chromosomes were successively showed in PKD inhibition groups. Green,α-tubulin; blue, DNA; Bar = 5 μm. (**B**) The percentage of spindle/chromosome defects were recorded in control and CID755673-treated oocytes. (**C**) Protein levels of p-MAPK in control and CID755673 treatment oocytes were determined by western blotting. Data are presented as mean ± s.d. from at least three independent experiments. **, significant difference (*P* < 0.01). ***, significant difference (*P* < 0.001).

Since proper spindle formation is critical for the polar body extrusion during oocyte maturation, we assessed spindle morphology after PKD inhibition. As shown in Figure 2A, most oocytes exhibited a typical barrel-shaped spindle apparatus and well-aligned chromosomes on the metaphase plate in the control group; however, it displayed disorganized spindles with severely misaligned chromosomes in CID755673 treatment oocytes. The statistical data of abnormal spindle also confirmed this: As shown in Figure 2B, compared with 8.80 ± 7.39% (n = 154) abnormal spindle in control oocytes, up to 68.70 ± 4.98% (n=112, *P* < 0.001) CID755673-treated oocytes displayed disorganized spindles and misaligned chromosomes. We next explored the possible regulatory mechanism for the roles of PKD on spindle formation in oocytes. Our results showed that the expression of p-MAPK was remarkably reduced after CID755673 treatment. The relative intensity of p-MAPK also confirmed this (1 versus 0.36 ± 0.25, *P* < 0.01; Figure 2C). Taken together, our data imply that PKD is involved in the regulation of MAPK phosphorylation for meiotic spindle organization during porcine oocyte meiosis.

### Inhibition of PKD family affects p-cofilin-mediated actin assembly in porcine oocytes

**Figure 3:**
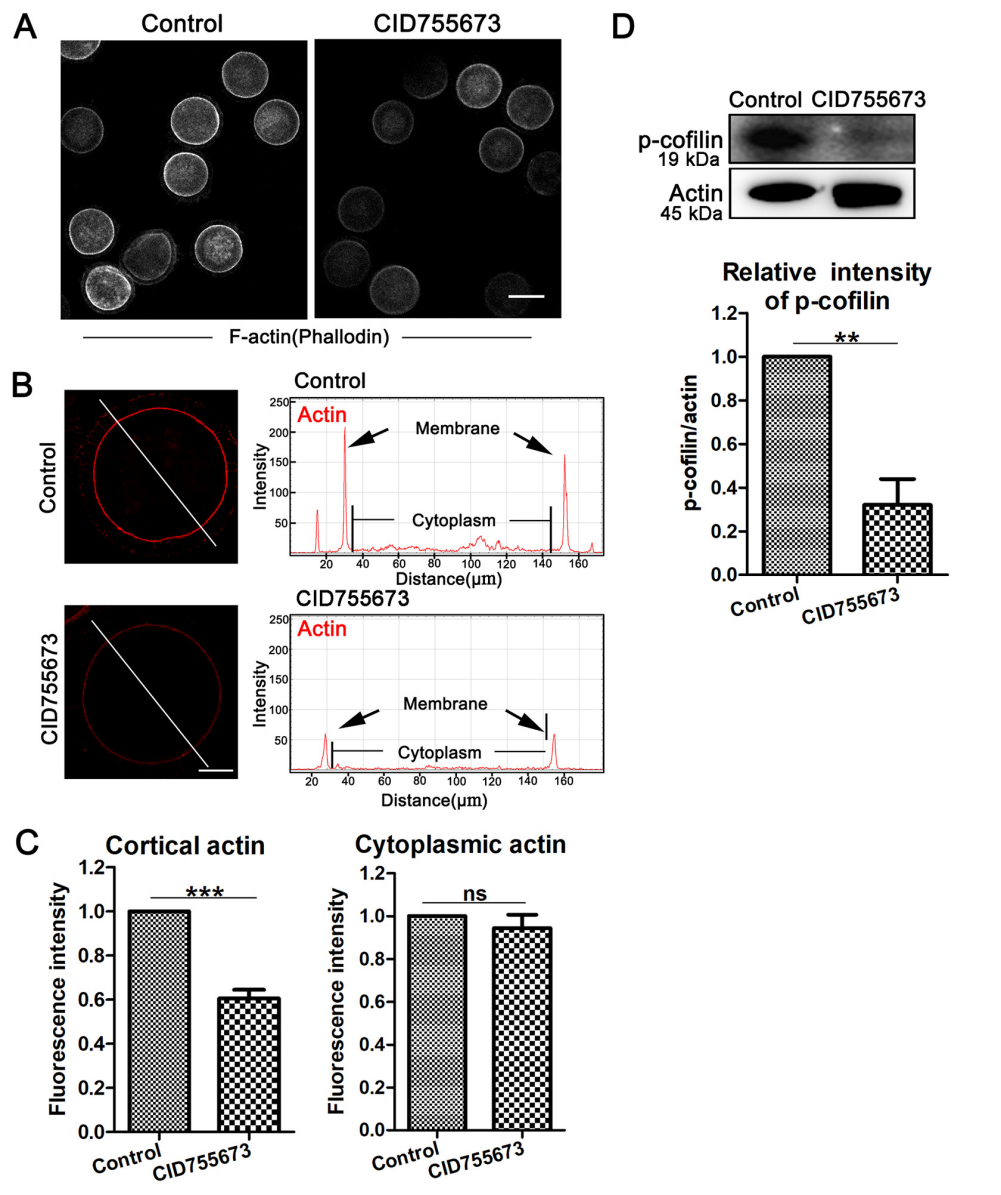
**Effects of PKD inhibition on cortical actin assembly and p-cofilin expression during meiosis.** (**A**) Representative images of actin distribution in the control and CID755673 treatment groups. White, actin, bar = 100 µm. (**B**) Fluorescence intensity profiling of actin in the left graphs. Lines in the same direction were drawn through the oocytes, and actin intensities were quantified along these lines. The black arrow in right graphs indicated actin intensity in membrane of oocyte. ZEN Blue Lite software was chosen to perform the analysis. Red, actin, bar = 30 μm. (**C**) Quantification immunofluorescence intensity levels of actin at the cortex and in the cytoplasm in the control and CID755673 treatment oocytes. Fluorescence intensities were analyzed using ImageJ software. (**D**) Protein levels of p-cofilin in control and CID755673 treatment oocytes were determined by western blotting. Data are presented as mean ± s.d. from at least three independent experiments. **, significant difference (*P* < 0.01). ***, significant difference (*P* < 0.001).

In addition, actin filaments are also the general power for the extrusion of polar body in oocytes. We also assessed the arrangements of the actin filaments in oocytes after CID755673 treatment. In our study, F-actin staining with phalloidin was applied, as shown in Figure 3A, the accumulation of actin signals at the cortical region significantly decreased in CID755673-treated oocytes compared with the controls, which was also confirmed by the actin fluorescence intensity analysis from the lineation (Figure 3B). Furthermore, quantitative analysis of cortical actin fluorescence intensity was also consistent with the above results, showing that inhibition of PKD substantially decreased actin intensity at the cortex of oocytes (1 versus 0.61 ± 0.08, n = 36; *P* < 0.001; Figure 3C). While there was no difference for the actin fluorescence intensity in the cytoplasm of CID755673 treatment oocytes compared with the control groups (Figure 3C). Actin assembly defects drove us to further explore the underlying regulator of PKD in porcine oocytes. As shown in Figure 3D, compared with the controls, the expression of p-cofilin was remarkably reduced after PKD inhibition. Quantitative analysis of p-cofilin band intensity also confirmed this (1 versus 0.32 ± 0.24; P < 0.01; Figure 3D). Therefore, these data suggest that PKD may be involved in cofilin phosphorylation for actin arrangement during porcine oocyte meiosis.

### Expression of PKD1 decreases in postovulatory aging porcine oocytes

**Figure 4:**
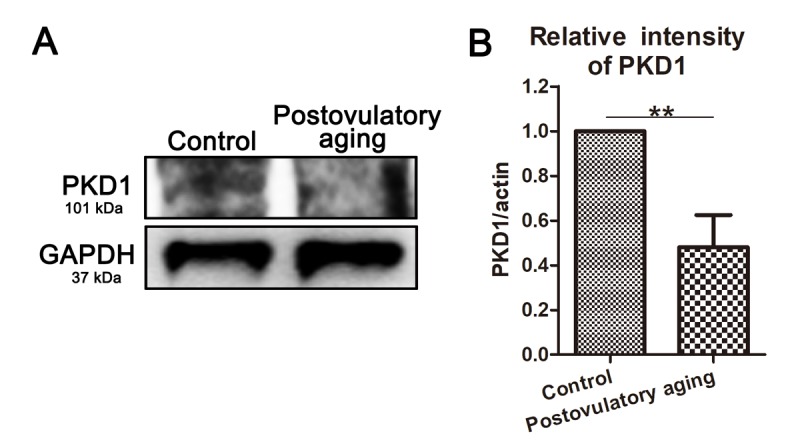
**PKD1 expression decreases in aged oocytes.** (**A**) Protein levels of PKD1 in control and postovulatory aging porcine oocytes were determined by western blotting. Rabbit polyclonal anti-PKD1 antibody was adopted. (**B**) Quantitative analysis of PKD1 expression in control and aging groups. Data are presented as mean ± s.d. from at least three independent experiments. **, significant difference (*P* < 0.01).

In this study, we also performed western blot analysis of oocytes to explore whether the expression of PKD family were related with oocyte aging using the postovulatory aging oocytes. And we found that PKD1 expression decreased in postovulatory aging oocytes compared to controls (Figure 4A). Moreover, quantitative analysis of band intensity also confirmed this (1 versus 0.48 ± 0.29; *P* < 0.01; Figure 4B). Thus, these results suggest that such a reduction of PKD expression might be related with cytoskeleton defects in aged porcine oocytes.

## DISCUSSION

In the present study, we explored the functions of PKD during porcine oocyte meiotic maturation. We showed that PKD was critical for meiosis resumption, spindle organization and actin microfilament assembly, which eventually affected PBI extrusion during porcine oocyte maturation.

We first showed that PKD expressed in porcine oocytes, indicating that PKD family may play roles during porcine oocyte meiotic maturation. To verify our hypothesis, by using a specific inhibitor CID755673, we disrupted PKD activity and assessed the polar body extrusion as well as the meiotic procession. Previous study indicated that the treatment with 5 μM CID755673 resulted in significant inhibition of NK cells [22]. Additionally, the phosphorylation levels of PKD were completely blocked at 50 μM CID755673 treatment [23]. Thus, in the current study four different concentrations of 5 μM, 10 μM, 20μM and 50 μM were applied to investigate the roles of PKD in oocytes. Our results showed a significant failure of the polar body extrusion after PKD inhibition when CID755673 were raised to 10 μM; moreover, 50 μM CID755673 strongly blocked meiosis resumption. These findings were expected because PKD3 knock-down in mouse embryonic fibroblast cells disrupted the completion of proper cell cycle, thus reduced their proliferation rate [24]. In addition, it was proposed that other PKD inhibitor, SD-208 and CRT006610 induced G2/M cell cycle arrest and suppressed the proliferation of cancer cell [25,26]. Therefore, we suggest that PKD may positively affect polar body extrusion and meiotic process of porcine oocytes.

We then analyzed the causes for the defects of polar body extrusion after PKD inhibition. Our results showed abnormal spindle morphologies with misaligned chromosomes in porcine oocytes after PKD inhibition, indicating a critical role for PKD in spindle formation and chromosome alignment during porcine oocyte meiosis. Similar results were found for other protein kinases in mitosis, showing that Polo-like kinase 1-mediated Kindlin-1 phosphorylation ensuring mitotic spindle assembly and cellular survival [27]. The functions of protein kinases on meiotic spindle apparatus are also proposed. It has been shown that PKCs play important roles in regulating spindle organization in mouse eggs [28]. Polo-like kinase 4 and Aurora kinase A cooperate in the initiation of acentriolar spindle assembly during oocyte meiosis [29]. Thus, our results indicate that similar with other protein kinases, PKD family is also involved in spindle organization, which regulates polar body extrusion during porcine oocyte maturation. Furthermore, to analyze the regulatory approach of PKD family on spindle formation, we examined MAPK phosphorylation levels, which are critical for spindle formation and stability in oocytes [30]. Our results showed the expression level of phosphorylated MAPK reduced significantly after PKD inhibition. In other models PKD has also been linked to regulate the activation of MAPK family external signal-regulated kinases (ERK1/2) signaling pathway for endothelial cell proliferation [31]. Overall, we conclude that PKD positive modulates the level of p-MAPK, thereby contributes to spindle formation during porcine oocytes meiosis.

Moreover, the defects of polar body extrusion are probably caused by abnormal actin dynamics, including cortical and cytoplasmic actin filaments [32,33]. We then assessed actin distribution, and we found a significant decrease of cortical actin expression after PKD activity inhibition, which suggested the involvement of PKD in actin assembly during porcine oocyte meiosis. Our results were supported by the previous study showing that PKD was pivotal for F-actin stabilization and remodeling in mitotic cells [20,34]. It has been proposed that the prominent role of PKD on stabilizing F-actin probably was achieved through multiple downstream effectors. PKD1 phosphorylated and modulated the activity of cortactin, a key regulator of actin dynamics, and was responsible for actin assembly [35]. It was also reported that PKD participated in RhoA signaling pathway for regulating cofilin-mediated F-actin reorganization [21]. In present study, we found that p-cofilin expression was greatly reduced after PKD inhibition. Thus, our data demonstrate that PKD plays conserved roles on the regulation of cofilin-mediated actin assembly for porcine oocyte maturation.

Age-related decrease in fertility becomes a hallmark of animal development and human reproduction [36,37]. The aged eggs of compromised developmental competence is pivotal characterized by the disruption of subcellular structures like spindle/chromosome anomalies and actin distribution defects [38]. Since PKD inhibition is also accompanied by spindle abnormality and actin distribution disorganization, we wondered if the PKD was involved in the aging-associated oocyte meiotic defects. Our results showed the decreased levels of PKD1 in postovulatory aging porcine oocytes, indicating a possible involvement of PKD in oocyte developmental competence during postovulatory aging.

In summary, our data indicate that PKD phosphorylates MAPK for spindle organization and cofilin for actin assembly during porcine oocyte meiotic maturation. Meanwhile, the decreased PKD1 might be one potential reason for the cytoskeletal defects in aged oocytes.

## MATERIALS AND METHODS

### Antibodies and chemicals

Rabbit polyclonal anti-PKD1 antibody (#SAB4502371), mouse monoclonal anti-α-tubulin-FITC (F2168) antibody and Phalloidin-Atto 590 (93042) were purchased from Sigma (St. Louis, MO, USA). Rabbit monoclonal anti-PKD antibody (#2052), rabbit polyclonal anti-phospho-MAPK antibody (#4370), rabbit monoclonal anti-p-cofilin antibody (#3313), mouse monoclonal anti-β-actin antibody (#3700), rabbit monoclonal anti-GAPDH antibody (#2118) were purchased from Cell Signaling Technology (Danvers, MA, USA). CID755673 was from Selleck (Houston, TX, USA). All other chemicals were purchased from Sigma (St. Louis, MO, USA), unless otherwise stated.

### COCs collection and IVM

All procedures with animals were conducted according to the Animal Research Ethic Committee guidelines of Nanjing Agriculture University, China. The experimental protocols were approved by Nanjing Agriculture University Animal Research Ethic Committee. Porcine ovaries from prepubertal gilts were collected and transported from the local abattoir to laboratory in sterile physiological saline (0.9% NaCl) containing 800 IU/ml of gentamicin within 2 h. By aspiration with a 20-gauge needle attached to a 10 ml disposable syringe, cumulus-oocyte complexes (COCs) were harvested from 3-6 mm antral follicles. Oocytes with intact and compact cumulus cells and a uniform ooplasm were selected from the subnatant precipitate of follicular fluid for study. After four times rinsing with in vitro maturation modified medium 199 containing 0.1% (wt/vol) polyvinyl alcohol (PVA), 3.05 mM D-glucose, 0.91 mM sodium pyruvate, 0.57 mM cysteine, 50 μg/ml of streptomycin, 75 μg/ml of penicillin, 10 IU/ml of hCG, 10 IU/ml of PMSG, 10 ng/ml of epidermal growth factor (EGF) and 10% pFF. A group of sixty COCs were cultured in 500 μl of IVM medium covered with 200 μl mineral oil in each well of a four-well dish (Nunc, Roskilde, Denmark) at 38.5°C in a humidified atmosphere of 5% CO_2_ incubator.

### CID755673 treatment *in vitro*

A specific PKD family inhibitor, CID755673 was employed to inhibit the activity of intracellular PKD during oocytes meiosis. COCs were divided into two groups, as follows: (i) non-treated (control) group; (ii) treatment group with CID755673. To evaluate the effects of CID755673 on COCs viability and oocyte maturation, a 50 mM stock solution was prepared in DMSO and then diluted to final concentration of 5 μM, 10 μM, 20 μM and 50 μM in IVM medium. Control oocytes were treated with the same concentration of DMSO. After culture with PKD inhibitor, oocytes were prepared for phenotypic analysis.

### Assessment of nuclear maturation and meiotic progression

Oocyte maturation is judged by both cumulus cells expansion and polar body extrusion. After 44 h culturing, cumulus cells expansion degree was assessed subjectively as fully expanded (all cumulus cells were loosened), partially expanded (the outer layer of cells was loosened) or not expanded (none of cumulus cells were loosened) under a stereomicroscope. Subsequently, for assessing the polar bodies emission, oocytes were then separated from surrounding cumulus cells completely by pipetting gently with a fine-bore pipette in 0.02% (w/v) hyaluronidase (in TCM-199), a group of denuded oocytes were gathered together and then rotated one by one with a thin sealed glass needle under a Olympus IX53 inverted microscope (Olympus Corp., Tokyo, Japan) (100X), and those with polar bodies were judged as matured oocytes. For determining the meiotic progression, denuded oocytes were stained with Hoechst 33342 and observed under a Zeiss LSM 700 META confocal system. Oocytes being in the germinal vesicle stage (GV) were considered immature, oocytes from GVBD-MII stage were considered to be restore maturational progression.

### Immunofluorescence staining and confocal microscopy

The denuded oocytes were fixed with 4% (w/v) paraformaldehyde (in PBS) for 30 min and then permeabilized with 1% Triton X-100 (in PBS) for 8-12 h at room temperature. To suppress nonspecific binding of IgG, oocytes were blocked in 1% bovine serum albumin (BSA)-supplemented PBS for 1 h at room temperature. For evaluation of spindle configuration, oocytes were incubated with anti-α-tubulin-FITC antibody (1:400) overnight at 4°C. For actin staining, oocytes were labeled with Phalloidin-TRITC (5 μg/ml in PBS) for 1 h at room temperature. After washing three times, oocytes were stained with Hoechst 33342 (10μg/ml in PBS) for 15 min. Finally, samples were mounted on glass slides, and observed under a confocal laser-scanning microscope (Zeiss LSM 700 META; Germany).

### Protein extraction and Western blot analysis

After in vitro culture for 27 h, a total of 150 porcine oocytes were collected and lysed in 2×Laemmil SDS sample buffer (Bio-Rad, Hercules, CA, USA). Proteins were boiled for 10 min at 100°C. After cooling and centrifugation, samples were stored at -20°C. Protein extracts were separated using NUPAGE 12% Bis-Tris Precast Gel (Thermo Fisher, Waltham, MA, USA) and electrically transferred onto polyvinylidene fluoride (PVDF) membranes (Millipore, Billerica, MA). Membranes were then blocked in Tris-buffered saline containing 0.1% Tween-20 (TBST) and 5% (w/v) BSA for 1 h at room temperature, and subsequently incubated with a rabbit monoclonal anti-PKD antibody (1:1000), rabbit polyclonal anti-phospho-MAPK antibody (1:1000), rabbit monoclonal anti-p-cofilin antibody (1:1000) and rabbit polyclonal anti-PKD1 antibody (1:500) at 4°C overnight. After washing three times in TBST (10 min each), membranes were incubated for 1 h with a 1:5000 dilution of HRP-conjugated goat anti-rabbit IgG. Finally, chemiluminescence was performed with ECL Plus Western Blotting Detection System (Tanon-3900, China). Equal protein loading was confirmed by the levels of actin (1:2000) or GAPDH (1:2000).

### Statistical analysis

All experiments were performed at least three times. The mean and standard deviation were calculated by Student’s t-test to compare statistical significance of test materials against the control. All analyses were performed using GraphPad Prism. Results of *P* < 0.05 were considered statistically significant (differences *P* < 0.05 denoted by *, *P* < 0.01 denoted by ** and *P* < 0.001 denoted by ***). Values are presented as means ±s.d.
